# The Level of Self-Esteem and Sexual Functioning in Women with Idiopathic Scoliosis: A Preliminary Study

**DOI:** 10.3390/ijerph120809444

**Published:** 2015-08-12

**Authors:** Jacek Durmała, Irmina Blicharska, Agnieszka Drosdzol-Cop, Violetta Skrzypulec-Plinta

**Affiliations:** 1 Chair and Department of Rehabilitation, School of Health Sciences in Katowice, Medical University of Silesia, ul. Medyków 12, 40-752 Katowice, Poland; E-Mails: reh@gcm.pl (J.D.); iblicharska@sum.edu.pl (I.B.); 2 Chair of Woman’s Health, School of Health Sciences in Katowice, Medical University of Silesia, ul. Medyków 12, 40-752 Katowice, Poland; E-Mail: skrzypulec-plinta@o2.pl

**Keywords:** sexual dysfunction, psychological test, posterior trunk symmetry index, female sexual function index

## Abstract

A person’s image, which is determined through physical appearance, considerably affects self-esteem developed from early childhood. Scoliosis causes multiple trunk deformations that can affect a person’s perception of the body. The aim of the study was to analyze the impact of scoliosis dimension and the degree of trunk deformation on the level of self-esteem and sexual functioning in women with idiopathic scoliosis. Thirty-six women diagnosed with idiopathic scoliosis were recruited to a prospective, double-blind, randomized controlled trial. The subjects were divided into two groups depending on the value of the Cobb angle. The level of self-esteem was determined by means of the Rosenberg Self-Esteem Scale (SES), whereas the sexual functioning was assessed via the Female Sexual Function Index (FSFI). The trunk deformations were specified with the Posterior Trunk Symmetry Index (POTSI). A statistically significant correlation was proved between the amount of points received in the Rosenberg scale evaluation and the POTSI index in Group A (*R* = −0.56, *p* = 0.04). Subjects with smaller deformations within the coronal plane had a higher level of self-confidence. The trunk asymmetries in the coronal plane may have a negative effect on women with scoliosis and their self-appraisal.

## 1. Introduction

Self-esteem is defined as a perception of the inner-self, determining a favourable relation to oneself. Rosenberg specifies it as positive or negative attitude of oneself. The concept itself is more complex and multidimensional. Its different types are usually not distinguished in Anglo-Saxon literature. Instead, they are all named as “global self-esteem”. That construct, which is based on the subjective perception of self-esteem, has a relatively permanent state in adults [1,2]. High self-esteem influences the level of life satisfaction. It is connected with personality traits that decide on experiencing positive emotions, as well as the easier tendencies of becoming irritated or aggressive. Global self-esteem is highly connected with dejectedness whereas low self-esteem is specified as a neurotic manifestation [3,4]. Horney points out the genesis of these disorders in childhood. The experiences may cause a sense of worthlessness, which is reflected in the person’s functioning in society, undertaken tasks, or their own perception of attractiveness [1,4]. Self-appraisal may be treated twofold: as a trait that is largely genetically conditioned and dependent on current events, or as a state mirrored in a level of social approbation felt in a particular moment [1,2].

Next, a multidimensional problem that can affect female functioning is sexual dysfunction. It has a negative influence on women’s quality of life, and can also influence the partner’s quality of life and the mental health of other family members. Negative body image is one of the factors that affects sexual function [5].

Many authors tried to undertake the analysis of self-appraisal or psychosocial development of people with scoliosis. However, the coverage of facts is usually contradictory and it does not show the appropriate standard. Moreover, the specialized literature is not exhaustive enough as far as sexual disorders are concerned in the already mentioned group of women.

Scoliosis is a three-dimensional spine deformity that causes abnormalities and morphological changes of the body, notably shown in the coronal plane [6]. The critical moments of postural genesis, in which a variety of deformations appear, are the so-called periods of rapid growth. In those stages, the child’s lifestyle changes. There are also modifications in the psyche and personality. Children with physical disorders do not accept their appearance since they notice their otherness. Those individuals may sense frustration and have low self-esteem [7,8]. It often happens in cases of people with scoliosis that are frequently exempt from loco-motor activities or those that wear special orthopaedic braces. In many instances, the treatment’s priority for a female patient or her parents is the improvement of the outer signs of deformation (the trunk symmetry, the lack of protruding scapulae, *etc.*), as opposed to the actual correction of spine deformations.

The purpose of this research was to analyze the impact of scoliosis dimension (the Cobb angle) and the degree of trunk deformation (based on the Posterior Trunk Symmetry Index) on the level of self-esteem as well sexual functioning in young women with idiopathic scoliosis (who up to that moment were subjected to a conservative treatment). Therefore, an attempt was undertaken at determining the frequency of sexual dysfunction in women with scoliosis and the domain predominantly affected by disorders.

## 2. Experimental Section

### 2.1. Methods

Between November 2012 and June 2014, the female patients of the Rehabilitation Department were enrolled in a cross-sectional cohort study after fulfilling the following inclusion criteria (Table 1):

**Table 1:** The main eligibility criteria for study group.

Inclusion	Exclusion
Sex: women	
Age: ≥18 years old	
Diagnosed with adolescent idiopathic scoliosis (discovered from the age of 10 years before the end of growth)	Non-idiopathic spine deformation or diagnosed before 10 years old or after the end of growth
Treatment according DoboMed method in connection with Cheneau brace	Non-braced patients, after surgery intervention, or treated by any method other than DoboMed method
Women who are sexually active	Women who have never had sexual intercourse
Women without additional diseases or damage that can affect sexual functioning	Women with additional diseases or damage that can affect sexual functioning

### 2.2. Participants

The study group consisted of 36 sexually active women, aged *x* = 20.7 ± 1.88 with diagnosed adolescent idiopathic scoliosis, treated by means of kinesiotherapy according to the DoboMed method in connection with the Cheneau brace. The subjects were divided into two groups depending on the Cobb angle dimension within the primary curvature. The Cobb angle is used to quantify the magnitude of spinal deformities. In the case of scoliosis, it is used to measure the coronal plane deformity on antero-posterior plane radiographs. Group A was formed by women with scoliosis below 30°, whereas Group B by those with more than or equal 30°. Angular parameters were determined on the basis of a current X-ray picture.

Patients were hospitalized and they went through an intensive stationary scoliosis therapy by means of the DoboMed method. Moreover, they were treated with a scoliosis brace. None of the tested women underwent a surgical correction of scoliosis. Table 2 below presents the tested group characteristics including anthropometrical measurements and the average angular value of primary curvature. The numerical amount of the respective groups is: 19 patients (Group A) and 17 (Group B).

**Table 2:** The characteristic of the study group.

	Average Measure and Standard Deflection		Scope (Min–Max)		*p*
	A (Cobb < 30°)	B (Cobb ≥ 30°)	A	B	
The Cobb Angle (°)	21.8 ± 5.77	44.2 ± 10.26	12–29	30–66	0.000001
Age (years)	20.4 ± 1.78	21.0 ± 2.0	18–24	18–24	0.28
Body weight (kg)	53.9 ± 9.58	57.4 ± 10.33	43–87	40–73	0.20
Height (m)	1.67 ± 6.4	1.67 ± 6.93	1.58–1.78	1.55–1.78	1.0
BMI (kg/m^2^)	19.1 ± 2.74	20.3 ± 2.8	15.9–28.4	16.3–25.6	0.18

### 2.3. Variables

In order to evaluate the women’s self-appraisal, the Rosenberg Self-Esteem Scale (SES) was applied (in Polish publications discussed by Dzwonkowska, Lachowicz-Tabaczek, and Laguna) and it enabled us to measure the general level of self-esteem considered a relatively constant trait as opposed to a merely temporary state [1]. The questionnaire is a one-dimensional, standardized tool that evaluates the relatively continual disposition comprehended as a conscious attitude towards the inner-self. It is characterized by a high standard of reliability and accuracy. The scale is composed of 10 diagnostic statements and the subject’s task is to specify to what extent she agrees with each proposition. Replies are provided according to a scale of four possible answers, where 1 pertains to “I strongly agree”. On the other hand, answer 4 states “I strongly disagree”. One part of those statements comprises positive opinions, such as “I am self-satisfied”. The other part concerns the success in life, the greater self-respect anticipation, and depreciation of oneself as a totally valuable person. The response time was unlimited. The average time was about two minutes. The total amount of points constituted the final result that was the general index level of self-esteem. The higher amount of points is ascribed to the answer indicating greater self-esteem. The scope of the points possible to acquire ranges from 10 to 40 [1,2].

For the evaluation of sexual activity, the FSFI (Female Sexual Function Index) questionnaire was used; it was composed of 19 questions about sexual experiences within the last four weeks. The analysis regards six domains: I—desire, II—arousal, III—lubrication, IV—orgasm, V—sexual satisfaction, VI—painful sensations connected with sexuality. The above-mentioned domains enable us to establish the occurrence of disorders in a particular sphere. Each domain is assigned to a particular number of questions. The points were summed up and multiplied by an adequate index assigned to a given sphere. The scope of points ranges from 0 to 6 points (the higher the value, the better the result in a given domain). The total amount of points from all the questions provides the final result. A score of 26.55 or lower indicates the occurrence of significant clinical sexual dysfunctions [9].

Women were also asked to fill in an authorial survey in regard to their own attractiveness assessment, as well as a personal opinion about whether scoliosis and its symptoms have an impact on their body perception and the quality of life. With the aid of an apparatus for a computer-based body posture examination (MORA 4G-projection moiré of 4th Generation), the spatial analysis and photogrammetric documentation of trunk morphology was effectuated. The apparatus includes, for instance, a projective and receptive device with a camera that is used for measuring pelvis and spine parameters. Its operation is based on photogrammetry, a discipline that is concerned with information acquisition about situating objects in space and in relation to each other. The construction itself combines the spatial analysis system MORA/ISIS (Integrated Shape Investigation System), as well as laboratories of movement based on markers. The location of eight anatomical points of the body in the program automatically assigns the value of the POTSI (Posterior Trunk Symmetry Index). This parameter was described by Suzuki. It enables a simplified analysis of deformation in a dorsal part of the trunk. Its value constitutes a sum of indices in the height difference of particular markers between the right and left side of the body, as well as asymmetry points of their distance from the central line. In the case of ideal symmetry, the POTSI equals 0 and it takes on higher amounts along with the development of deformation [10,11]. The parameters were compared with curvature value dimensions, specified on the basis of the Cobb methodology, that are determined on a current X-ray image in an upright position.

### 2.4. Design

Investigation procedures for the cross-sectional cohort study were performed by two independent teams of researchers. The first team evaluated morphological parameters of the trunk and deformities. The second team conducted a questionnaire survey, without acknowledging the type of scoliosis and the patients’ group affiliation. In addition, women were not informed about the research objective not to be influenced by means of their scoliosis type while providing answers in the questionnaire. The study itself obtained a positive opinion of the Bioethics Committee No KNW/0022/KB1/134/I/12 from the date: 23 October 2012.

### 2.5. Statistical Methods

The obtained results were analyzed by means of the Statistica v.10 program. The assessment of the normality of data was conducted with the Shapiro-Wilk test. To obtain statistical analysis of the parameters, we used non-parametric tests because tests for normality showed an abnormal distribution. The correlation between parameters was determined on the basis of the Spearman’s Test. For the comparison of parameters measured between groups, the Mann-Whitney U test was employed. On the other hand, for establishing differences in questionnaire answers, the Chi-squared test (χ^2^) was used. The value *p* < 0.05 was established for the level of statistical significance.

## 3. Results

The analysis of the results demonstrated the absence of statistically substantial discrepancies with regard to certain traits, *i.e.*, age, weight, height, BMI (*p* > 0.005). Moreover, the value of the POTSI parameter was not substantially different among the tested women (*p* = 0.20). The group with a lower value of the Cobb angle received the average POTSI result on the level of 20.55 ± 12.33, whereas Group B had 28.21 ± 16.03. The dependence between the POTSI value and the spine curvature angle in Group A (*R* = 0.42, *p* = 0.16) and in Group B (*R* = 0.07, *p* < 0.84) was not noticed.

**Figure 1:**
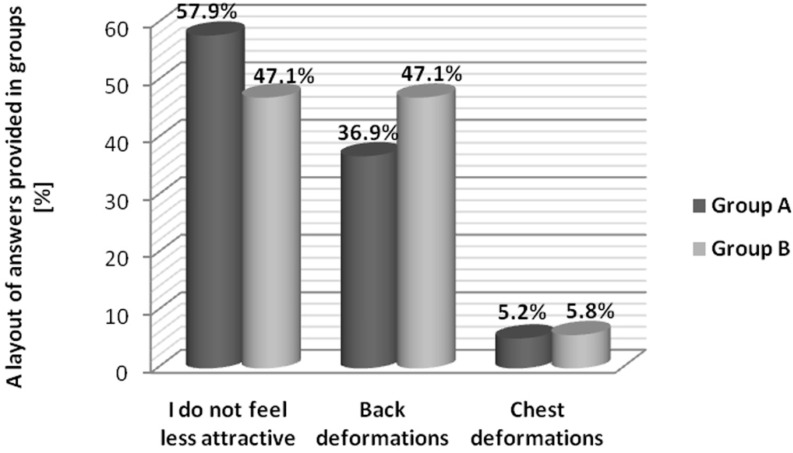
A representation of answers provided in groups.

The authorial survey included questions regarding attractiveness and a subjective assessment of the impact of the scoliosis on the function of associated deformations that influence personal sexual sensation. The majority of subjects did not feel less attractive by reason of their disorder. Moreover, 57.9% of women tested in Group A and 47.1% in Group B did not perceive the impact of spine curvature deformations on their own attractiveness. The diagram below shows the most frequent answers provided in the questionnaire (Figure 1).

The rest of the women from both groups frequently pointed out back deformations as a factor that has an impact on their lack of feeling attractive. Only few subjects selected chest deformations (the side and the front part of chest). The Chi-squared (χ^2^) test analysis shows evidence for the lack of typical discrepancies between group answers (*p* = 0.96).

In the group of women with a lower Cobb angle value, the average value result obtained via the Rosenberg Self-Esteem Scale was *x* = 22.4 ± 1.20, as opposed to Group B, where *x* = 23.25 ± 1.96. The result within the range 20–25 indicates the average level of self-esteem. The difference between groups (*p* = 0.16) is not statistically significant.

The table below (Table 3) depicts an average amount of points obtained from the particular domain analysis via the Female Sexual Function Index, as well as the test result showing variables between them and the angular degree of primary curvature.

**Table 3:** The score of the Female Sexual Function Index scale and the results of statistical analysis of correlation between the value of the Cobb angle in the primary curve and amount of points obtained in assessment of particular domains.

Domain	The Average Value of the Points		Correlation Test Cobb Angle *vs.* Domains	
	Group A	Group B	Group A	Group B
Desire	4.27 ± 0.89	4.45 ± 0.81	*R* = −0.4, *p* = 0.13	*R* = −0.1, *p* = 0.76
Arousal	4.77 ± 0.92	5.02 ± 0.71	*R* = −0.2, *p* = 0.50	*R* = −0.4, *p* = 0.18
Lubrication	5.65 ± 0.5	5.62 ± 0.59	*R* = −0.5, *p* = 0.11	*R* = −0.3, *p* = 0.29
Orgasm	4.49 ± 1.44	5.13 ± 0.74	*R* = −0.1, *p* = 0.78	*R* = −0.24, *p* = 0.45
Sexual satisfaction	5.08 ± 0.84	5.17 ± 0.71	*R* = −0.2, *p* = 0.53	*R* = 0.1, *p* = 0.77
Pain	4.49 ± 1.4	5.13 ± 0.91	*R* = −0.03, *p* = 0.91	*R* = 0.2, *p* = 0.57
Total FSFI	28.77 ± 4.15	30.53 ± 2.84	*R* = −0.3, *p* = 0.39	*R* = −0.04, *p* = 0.89

In order to estimate the impact of the value of the curvature degree on the evaluated domains, the correlation analysis was conducted. There were not any statistically significant relationship between value of the Cobb angle and particular spheres assessed in FSFI Scale. The representation of values was shown in Table 3.

Both groups obtained a result above 26.55 points, which indicates the absence of clinical disorders with respect to sexual functioning. In Group A, dysfunctions were observed in 15.4% of tested women. However, in Group B, there were none. Interestingly, patients with smaller angular values received fewer points, which indicates poorer sexual functioning in comparison to women with a Cobb angle above 30°. The difference between the groups in the total FSFI score is not statistically significant like in the case of the other domains (*p* > 0.05). In Group A, subjects frequently pointed out the problem with arousal. Group B received relatively better results. Nonetheless, sexual arousal was still assessed poorly.

A dependence was not observed between the result obtained with the Rosenberg Self-Esteem Scale and the angular value of primary scoliosis in both groups is (A: *R* = −0.36, *p* = 0.22; B: *R* = 0.18, *p* = 0.57). A statistically distinctive correlation was proven between the amount of points received on the Rosenberg scale evaluation and the values of the POTSI index in Group A (*R* = −0.56, *p* = 0.04). Subjects with smaller deformations within the coronal plane had higher self-esteem. In this group, the dependence between the POTSI parameter and the amount of points received in the FSFI (*R* = −0.68, *p* = 0.01) was noticed, which indicates better sexual functioning in women demonstrating more traits of trunk symmetry. However, a correlation between the SES scale and the result of the FSFI was not observed in both groups (A: *R* = 0.24, *p* = 0.42; B: *R* = 0.21, *p* = 0.52). There were no correlations between the POTSI index and the result of the Rosenberg Scale (*R* = 0.16, *p* = 0.68), or between the POTSI and FSFI scores (*R* = 0.26, *p* = 0.40) in group B.

## 4. Discussion

The study proved that significant clinical sexual disorders do not occur in women with scoliosis. The magnitude of scoliosis curvature does not affect evaluated domains. The correlation was marked between the degree of trunk deformation and the occurrence of sexual disorders for the group with smaller curves. We did not notice a relationship between sexual dysfunction and the Cobb angle. The cause of it can be, in fact, that the angular curvature value is not always connected with the magnitude of trunk deformation. Scoliosis is a disease that pertains to restraints in every day functioning and also contributes to trunk deformations. The visual correction of external deformation is an important issue in the rehabilitation of idiopathic scoliosis. The basic objective of comprehensive, conservative treatment is stopping scoliosis curvature at puberty or if it is possible, to reduce it. SOSORT (the International Scientific Society on Scoliosis Orthopaedic and Rehabilitation Treatment) experts proposed aesthetics as the first morphological goal of treatment. This aspect determines quality of life, disability, and the psychological well-being of patients with scoliosis more than value of the Cobb angle [6].

Self-esteem is discussed by scholars in various disciplines and spheres of knowledge. A wide range of research approaches can be found in specialized literature that evaluate the level of self-appraisal in women with various diseases or after surgical procedures, e.g., mastectomy, that affect the outer body appearance [12]. An attempt to establishing the level of self-esteem in women with scoliosis was frequently undertaken; however, the results are contradictory and its standard is not specified unambiguously. Various reports pertain to methods of treatment and their impact on self-esteem. Investigations conducted on a group of 247 women who underwent various therapeutic procedures, e.g., surgery, a scoliosis brace, indicated that there are no differences between them and the healthy population as far as the number of children or marital status is concerned, in comparison to the tested group [13]. In the research pertaining to the quality of life and self-appraisal in women with scoliosis, Danielsson denotes the impact of brace usage onto particular parameters. Subjects who were not treated using a brace perceived their bodies as less deformed [14]. Cheung proved a negative correlation between the time of scoliosis brace treatment and self-appraisal, which was not confirmed by Misterska [15,16,17].

The research was conducted in order to determine the possible factors of sexual disorders in women diagnosed with scoliosis. In the available specialized literature, there is a deficiency of data investigating sexual disorders not only in regard to the angular values of scoliosis, but also in regard to body deformations in this group of women. The present work is characterized by an innovative approach and it contains the preliminary analysis of the above-mentioned problem.

Unfortunately, in the present study, it was not possible to carry out an accurate analysis that included the time of brace treatment. The reason for that was a disagreement between the declared time of wearing the brace stated by parents and subjects, as well as observations from doctors (the clinic or hospital documentation) who prepare the prospective data analysis with regard to a brace condition, its set-up technique, and signs on a patient’s skin. In case of subjects and their earlier treatment by means of a brace, the patients’ declarations were not verified with any sensors. In orthoses that were used by them, for instance, thermal sensors were not applied because they were not universally available during the time of their treatment. Other limitations of the study were a small sample, and the fact that subjects were hospitalized, which can affect the reliability of the results. By virtue of the topic’s sensitivity and controversial points of view, women frequently refuse to take part in research that explores that sphere of their activity, which adds constraints to direct further evaluation to study groups including patients undergoing a long-term scoliosis treatment by means of kinesiotherapy and the Cheneau brace, so the obtained results do not concern untreated women with scoliosis or those after surgical intervention.

The research will be continued and developed with further conclusions. The research will be extended on a larger number of participants and on patients after the surgical treatment of scoliosis. The evaluation of sexuality and self-esteem will be supplemented by other psychological variables that have an impact on a low level of arousal in women with scoliosis.

Self-esteem as a way of perceiving one’s own body is conditioned by various factors. So far, it was not explicitly defined to what extent scoliosis and its therapeutic procedures can determine self-esteem. This study points out that the Cobb angle does not have a significant influence in the quality of life of patients, and confirms the importance of the improvement of aesthetics as an aim of treatment.

## 5. Conclusions

The curvature deformation in the tested group of young women with idiopathic scoliosis, who were treated by means of conservative methods in their development period, does not have an impact on their self-esteem and sexual functioning. The trunk asymmetries in the coronal plane are associated with women’s (scoliosis) self-confidence and sexual arousal. The evaluation, though, needs to be scrutinized and further investigated.
